# Hepatic Epithelioid Hemangioendothelioma Presenting as an Enlarging Vascular Lesion within the Spleen

**DOI:** 10.1155/2018/3948784

**Published:** 2018-04-12

**Authors:** Juliet A. Emamaullee, Klaudia Nowak, Marla Beach, Julinor Bacani, A. M. James Shapiro

**Affiliations:** ^1^Department of Surgery, University of Southern California, Los Angeles, CA, USA; ^2^Department of Anatomic Pathology, University of Alberta, Edmonton, AB, Canada; ^3^Department of Surgery, University of Alberta, Edmonton, AB, Canada

## Abstract

Epithelioid hemangioendothelioma (EHE) is a rare vascular neoplasm with variable malignant potential that most often presents within the liver. Many patients present with bilobar or extrahepatic disease, and the current treatment paradigm involves liver transplantation, with favorable long term results. Up to 25% of patients are diagnosed incidentally following imaging for other indications, and confirmation of diagnosis requires histologic analysis, as there are no classical imaging features to distinguish hepatic EHE (HEHE) from other solid hepatic lesions. Here we describe a case of microscopic HEHE that was diagnosed following splenectomy for an enlarging vascular tumor within the spleen. Due to the unexpected diagnosis of EHE within the spleen and coexisting but stable appearing liver hemangiomata, a left hepatic lobectomy was performed. Explant histology revealed benign hemangiomata and diffuse, microscopic HEHE. The patient ultimately underwent liver transplantation. HEHE can be a challenging diagnosis, and this case emphasizes that any enlarging vascular lesion, even within the spleen, should prompt a high index of suspicion for HEHE in the setting of known hemangiomata.

## 1. Introduction

Epithelioid hemangioendothelioma (EHE) is a rare vascular neoplasm that occurs more often in women than men and usually arises within the liver, but can also present within other tissues including lung, bone, or peritoneum [1]. The behavior of these tumors is variable, ranging from a relatively benign clinical course to rapidly progressive, metastatic disease [1–3]. More than 25% of patients are asymptomatic and diagnosed after incidental finding of a liver lesion [4]. The mainstay of treatment for hepatic epithelioid hemangioendothelioma (HEHE) is surgical resection or transplantation, and excellent long term outcomes have been reported [4, 5]. Typically, HEHE presents with bulky tumors, which may be multifocal and/or bilateral, and >30% of patients may present with extrahepatic disease [4]. In the present case report, we describe a patient with multiple benign, classical appearing hepatic hemangiomata and an atypical, enlarging vascular lesion within the spleen which lead to the diagnosis of microscopic HEHE and ultimately liver transplantation.

## 2. Case Report

**Figure 1:**
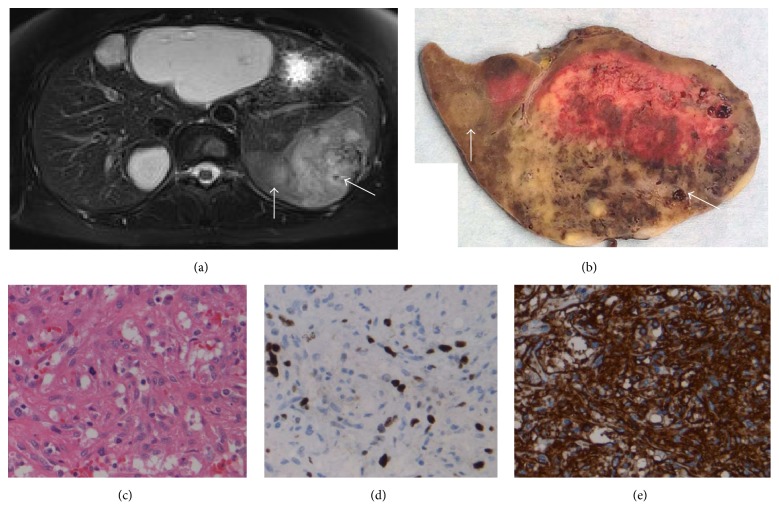
Epithelioid hemangioendothelioma arising within the spleen. (a) Axial MRI scan showing liver hemangiomata (white masses) and vascular splenic lesions. (b) Gross specimen following splenectomy. White arrows correspond to imaging findings in (a). (c) H&E staining demonstrated a highly cellular, atypical complex vascular lesion with rare mitotic figures. (d) MIB-1 staining illustrates a proliferation index of approximately 10%. (e) Immunophenotyping with CD31.

A 49-year-old woman with no significant past medical history presented to her family physician in 2014 with vague right upper quadrant abdominal pain. Her vital signs were stable, her abdominal exam was unremarkable, and she had no palpable organomegaly. Her hemoglobin, platelet count, white cell count, liver function, and renal function tests were all within normal limits. She underwent abdominal ultrasound, which demonstrated multiple vascular lesions within the liver, with the appearance of hemangiomata. Her abdominal pain had since resolved, but due to the ultrasound findings, she received an MRI of the abdomen. This study demonstrated multiple liver lesions with enhancement patterns consistent with hemangiomata. There was also a 4.6 × 3.1 × 2.8 cm vascular lesion within the body of the spleen that enhanced with features consistent of hemangioma, although it was less bright than the liver hemangiomata. She remained asymptomatic. These lesions were followed with serial MRI studies, and while the liver lesions remained stable in size and enhancement, follow-up imaging at two years demonstrated an interval enlargement in the splenic lesion to 7.1 × 5.0 × 6.3 cm, with four additional new nodules within the spleen (Figure 1(a), white arrows). The dominant lesion had heterogenous enhancement compared to the liver hemangiomata (white lesions within the liver in Figure 1(a)).

**Figure 2:**
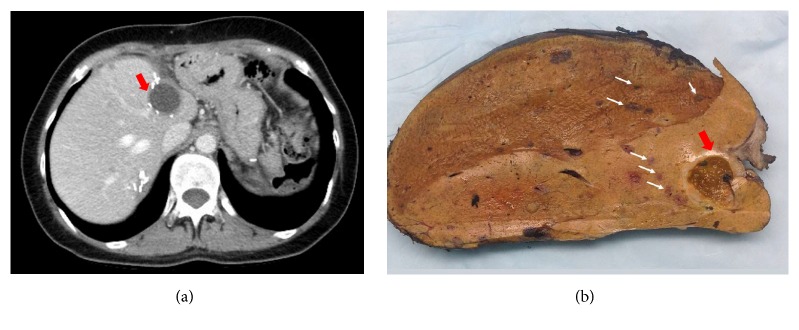
Epithelioid hemangioendothelioma in explant following liver transplantation. (a) Axial CT scan showing liver hemangioma (dark mass) and no obvious additional lesions. Red arrow indicates known residual hemangioma following left lobectomy. (b) Gross specimen following hepatectomy and liver transplantation. White arrows indicate multifocal EHE which was not apparent on preoperative imaging studies. Red arrow indicates corresponding lesion from (a), which demonstrated microscopic EHE within hemangioma on microscopic examination (not pictured).

Due to the increase in size, new nodules, and heterogeneous enhancement, she was referred for surgical resection. She underwent an uncomplicated open splenectomy in September 2016. Gross pathology demonstrated an 8.0 × 5.8 × 4.5 cm lesion with a mottled appearance and focal hemorrhagic tissue which nearly replaced the splenic parenchyma (Figure 1(b)), which was consistent with preoperative imaging (Figure 1(a); white arrows correlate to similar areas in gross specimen in Figure 1(b)). Histological assessment demonstrated a highly cellular, atypical complex vascular lesion with rare mitotic figures on H&E staining (Figure 1(c)). MIB-1 staining illustrates a proliferation index of approximately 10% (Figure 1(d)). Immunophenotyping with CD31 (Figure 1(e)) and CD34 (not shown) confirmed the presence of vascular endothelium. Taken together, the morphological and immunophenotypic findings are consistent with a diagnosis of epithelioid hemangioendothelioma (EHE). Given this diagnosis, and the previously identified hepatic vascular lesions, she underwent a left hepatic lobectomy and nonanatomic resection of a segment 8 lesion to rule out HEHE in November 2016. Final pathology showed benign hemangiomata in the areas observed on imaging and multifocal, microscopic EHE within the liver parenchyma which was not appreciated on multiple previous imaging studies. Based on these findings, she was referred for liver transplantation. She was carefully evaluated by our multidisciplinary team and referred to medical oncology for consultation. She underwent full body imaging including a CT chest, abdomen, and pelvis, as well as abdominal and pelvis MRI, which did not demonstrate any new lesions or evidence of extrahepatic disease. The collective opinion based on multidisciplinary assessment was to proceed with liver transplantation as the best option for R0 resection. She ultimately underwent liver transplantation in early 2017, with evidence of multifocal HEHE in the explanted liver (Figure 2). There were no concerning lesions, nodules, or lymph nodes noted at the time of transplant, and no malignant periportal lymph nodes were identified within the explant. She received induction immunosuppression with basiliximab and maintenance therapy with tacrolimus and mycophenolate mofetil, which is the standard of care for most patients in our center. She has had an uncomplicated postoperative course with no evidence of recurrent EHE on follow-up imaging, with more than one year of follow-up to date.

## 3. Discussion

HEHE can be challenging to diagnose and also be a management dilemma, particularly in patients with multiorgan involvement. The case reported here is unique in that it appears as though the primary tumor developed within the spleen, with microscopic liver involvement. Historically, patients with multifocal disease have a more aggressive course and may not be considered good transplant candidates, although more recent data suggests that, with metastasectomy, these patients can have good outcomes [4]. It was somewhat surprising to diagnose microscopic HEHE in this patient, who had stable benign hepatic hemangiomata on imaging over more than two years. There are no pathognomonic imaging characteristics for EHE, and thus the gold standard for diagnosis is microscopic analysis. Histological features of EHE include high cellularity, rare mitoses, and evidence of endothelial markers including CD31 and CD34 [1]. Our group has previously reported vascular endothelial growth factor (VEGF) expression in EHE as well [6]. The mainstay of treatment for EHE remains surgical resection or liver transplantation, with excellent long term disease-free recurrence in most patients, at least within the context of HEHE [2, 4–6]. The present case illustrates that any enlarging vascular lesion, even within the spleen, should be referred for surgical evaluation and resection as it may represent a unique presentation of HEHE.
